# Epidemiology of Cryptococcal Meningitis in the US: 1997–2009

**DOI:** 10.1371/journal.pone.0056269

**Published:** 2013-02-15

**Authors:** Vasilios Pyrgos, Amy E. Seitz, Claudia A. Steiner, D. Rebecca Prevots, Peter R. Williamson

**Affiliations:** 1 Laboratory of Clinical Infectious Diseases, National Institute of Allergy and Infectious Diseases, National Institutes of Health, Bethesda, Maryland, United States of America; 2 Center for Delivery, Organization and Markets, Healthcare Cost and Utilization Project, Agency for Healthcare Research and Quality, Rockville, Maryland, United States of America; 3 Section of Infectious Diseases, Department of Medicine, University of Illinois at Chicago College of Medicine, Chicago, Illinois, United States of America; Instituto de Higiene e Medicina Tropical, Portugal

## Abstract

Cryptococcal meningitis (CM) causes significant morbidity and mortality globally; however, recent national trends have not been described. Incidence and trends for CM-associated hospitalizations in 18 states were estimated using the Agency for Healthcare and Research Quality (AHRQ) State Inpatient Databases (SID) datasets for 1997 through 2009. We identified 30,840 hospitalizations coded for CM, of which 21.6% were among HIV-uninfected patients. CM in-hospital mortality was significant (12.4% for women and 10.8% for men) with a total of 3,440 deaths over the study period. Co-morbidities of CM coded at increased frequency in HIV-uninfected CM hospitalized populations included hydrocephalus and acute/chronic renal failure as well as possible predispositions including transplantation, combined T and B cell defects, Cushing’s syndrome, liver disease and hypogammaglobulinemia. Median hospitalization costs were significant for CM and higher for HIV-uninfected patients (16,803.01 vs. 15,708.07; p<0.0001). Cryptococcal meningitis remains a disease with significant morbidity and mortality in the U.S. and the relative burden among persons without HIV infection is increasing.

## Introduction

Cryptococcal meningitis (CM) is a disease with significant morbidity and mortality that afflicts both immunecompetent and immune compromised hosts. Globally, approximately 1 million AIDS-related cases of cryptococcosis occur annually with more than 600,000 associated deaths, predominantly within the developing world where anti-retroviral therapy is less available [1]. At the same time, within the developed world, AIDS-related opportunistic infections such as CM have declined significantly [2], although widespread use of immunosuppression therapy for transplant conditioning and cancer chemotherapy has increased the size of populations susceptible to opportunistic infections such as CM. In addition, an increased recognition of cryptococcosis in apparently normal hosts is highlighted by a recent outbreak of cases in the Pacific Northwest centered in Vancouver Island in British Columbia, Canada [3]. Patients who survive CM may have long-term sequelae related to their infection such as focal neurologic deficits, blindness, deafness, cranial nerve palsies and memory deficits, and may require prolonged therapy or experience disease relapses [4].

Prior to the AIDS epidemic, cryptococcal infection was reported in less than 500 patients globally in the decade of the 1950’s [5], rising to 338 confirmed cases per year within the U.S. alone in 1976 [6]. This was followed by an explosion in cases with the advent of the AIDS pandemic [7] and a precipitous decline with the introduction of highly active anti-retroviral therapy (ART) [8]. However, while important risk factor and management issues have been elucidated from detailed studies in single centers or within specific risk groups such as transplant recipients [2], [9], [10], no current nationally-representative data for the U.S. exist regarding the incidence of cryptococcosis in these diverse patient groups, particularly the HIV-uninfected. Thus, in an effort to better understand the burden of cryptococcal disease in the United States in the post-ART era, we used data from the State Inpatient Databases of the Agency for Healthcare Research and Quality (AHRQ) through an active collaboration to describe the incidence of hospitalizations associated with cryptococcal meningitis, the most serious manifestation of cryptococcosis, during a 13 year period 1997–2009, and including both HIV-infected and uninfected populations in the U.S. AHRQ belongs to the Department of Health and Human Services and has compiled state level hospital discharge information since 1988 as part of the Healthcare Cost and Utilization Project (SID; www.hcup-us.ahrq.gov). These data illustrate a complex epidemiology, with a moderate decline in HIV-related CM and a persistence of non-HIV CM. Given that non-HIV CM is becoming a more significant subpopulation in the U.S. CM patient burden, we also conducted a national analysis of CM co-morbidities in the HIV-uninfected population to better understand possible risk factors and complications within this heterogeneous population.

## Methods

### Data Source and Study Population

This analysis was performed using the State Inpatient Databases (SID). In 2010, SID encompassed approximately 95% of U.S. hospital discharges from 44 states. Our study period included the years 1997 through 2009 and was limited to the 18 states that reported continuously during this period, containing information from over 200 million discharges. These 18 states represent over 50% of the total US population. Data were extracted as described [11] from the intramural databases maintained by AHRQ using the International Classification of Diseases, Ninth Revision, Clinical Modification (ICD-9-CM) code for CM (321.0). Because the data was de-identified and not collected for research purposes by hospitals, the study was not considered human subject research by the National Institutes of Health Office of Human Subjects Protection.

The study population included all hospitalizations with a discharge diagnosis code for CM as either the principal diagnosis or any of 9 secondary diagnoses. This approach of using multiple secondary diagnoses was used to reduce bias due to secular trends in documentation and coding priorities as recently described in studies of pneumonia [12]. Hospitalizations with any discharge diagnosis of HIV/AIDS were identified using ICD-9-CM code 042. To calculate age-, sex- and state- specific deaths and estimates, we used age- and sex-specific census data, which were available from the U.S. census. We used the study midpoint year, 2003 for a population denominator when calculating rates over a period. The annual population was used as the denominator when calculating annual estimates. HIV incidences were determined for the 18 participating states using the CDC HIV incident database. Epidemiological information included age, sex, co-morbidities and in-hospital mortality, the latter as defined by Lindenauer et al. [12]. To describe the most common co-morbid conditions associated with CM in our study population (Table 1), we assessed diagnosis claims among persons with CM. We identified the most common co-morbid conditions as well as the conditions with known or suspected associations with CM. Some co-morbid conditions were grouped. For example, hematological malignancies were grouped because none showed a strong relationship individually and non-tuberculous mycobacterial disease (NTM) was grouped with TB to improve its positive predictive value due to previous studies showing ambiguities introduced from inclusion of patients being evaluated for TB, found on subsequent culture to have non-tuberculous mycobacteria [13]. Relative prevalence for each co-morbidity was calculated as the ratio of the frequency of a given ICD-9 diagnosis among CM patients in 2009 compared with the overall proportion of the same diagnostic code among all hospitalizations for the same period. Significance of the relative prevalence was determined, adjusting for multiple comparisons as described below.

**Table 1:** Relative incidence of selected co-morbid conditions among CM hospitalizations compared to all-cause hospitalizations.

Diagnosis	Diagnosis Codes	CM related Hospitalizations (%)	All Hospitalizations (%)	P value	Relative Prevalence (95% CI)
Acute Renal Failure	584, 580.81	296(28.5)	2,119,041 (6.4)	<0.0001	5.8 (5.1–6.7)
Chronic Renal Failure	585, 582.9	149(14.3)	3,225,748(9.7)	<0.0001	1.6 (1.3–1.8)
Hydrocephalus	331.3, 331.4	117(11.3)	81,319(0.3)	<0.0001	51 (42–62)
Solid malignancies	154.1,155.0,155.2,162.9,173.3,174.3,174.8,174.9, 176.9, 185, 188.9, 191.5, 191.6, 191.8, 196.9, 197.0, 197.5, 197.7,198.3, 198.5, 198.7	68(6.5)	1,611,335(4.9)	0.01470	1.4 (1.1–1.8)
Hematopoietic malignancies	200.10, 200.18, 200.30, 200.50, 200.51, 200.84, 201.90,201.98, 202.00, 202.10, 202.40, 202.80, 202.83, 203.00,204.00, 204.10,204.11,204.12, 205.00, 205.10, 208.90,237.5, 238.6, 238.71, 238.75, 238.79	141(13.6)	567,354(1.7)	<0.0001	9.0(7.5–10.8)
Liver failure	572.2	19(1.8)	100,502(0.3)	<0.0001	6.1(3.9–9.6)
Liver cirrhosis	571.2, 571.5	38(3.7)	441,081(1.3)	<0.0001	2.8(2.0–3.9)
Leukopenia	284.1, 288.50, 288.51, 288.00	73(7)	453,355(1.4)	<0.0001	5.4(4.2–6.9)
T-cell Defect	279.1	10(1)	3,624(0.01)	<0.0001	88(48–165)
Hypogammaglobulinemia	279.02, 279.00	12(1.2)	10,643(0.03)	<0.0001	36(21–64)
Immunodeficiency NOS	279.3, 279.9	8(0.8)	7,315(0.02)	<0.0001	35(18–70)
Combined B- and T-cell defects	279.2	2(0.2)	1,075(0)	<0.0001	59(15–237)
Cushing’s Syndrome	255.0	8(0.8)	14,392(0.04)	<0.0001	25(12–49)
SLE	710.0, 695.4	27(2.6)	162,061(0.5)	<0.0001	5.4 (3.7–8.0)
Sarcoidosis	135	19(1.8)	63,883(0.2)	<0.0001	9.6 (6.1–15.2)
RA	714.0	12(1.2)	401,462(1.2)	0.97000	0.95 (.054–1.7)
Ankylosing Spondylitis	720.0	3(0.3)	12,865(0.04)	0.00100	7.5(2.4–23)
Dermatomyositis	710.3	3(0.3)	5,725(0.02)	<0.0001	17(5.4–1.7)
Autoimmune Hepatitis	571.42	3(0.3)	9,327(0.03)	<0.0001	10(3.3–32)
Kidney transplant	996.81	60(5.8)	56,178(0.17)	<0.0001	36(28–47)
Pancreatic transplant	996.86	4(0.4)	2,497(0.01)	<0.0001	51(19–136)
Viral hepatitis	070.30, 070.32, 070.71,070.54, 070.70	45(4.3)	578,276(1.75)	<0.0001	2.6(1.9–3.4)
Oral candidiasis	112.0	30(2.9)	201,210(0.61)	<0.0001	4.9(3.4–7.0)
Invasive Candidiasis	112.4, 112.5	6(0.6)	26,512(0.08)	<0.0001	7.3(3.3–16.2)
Mycobacterial Infections	013.0, 011.9, 015.0, 031.0	10(1)	19,287(0.06)	<0.0001	17(8.9–31)

Note: SLE =  Systemic Lupus Erythromatosus; RA = Rheumatoid Arthritis; NOS =  Not Otherwise specified.

Total CM related hospitalizations for the 2008–2009 period: 1,039. Total All hospitalizations for 2008–2009∶33,102,694.

### Statistical Analysis

Associations among categorical variables were assessed using the Chi-square test (survival comparisons, comparisons of men with disease, among all men, compared to women with disease, among all women) and the Wilcoxon test was used for non-parametric comparisons, with statistical significance assessed at p<0.05. Poisson regression was used to estimate trends in hospitalizations and in-hospital mortality, using the Annual Percent Change (APC) to quantify the rate. Significance of relative prevalence of co-morbidities using Chi-square was determined as p<0.0001, to adjust for multiple comparisons (200 ICD-9 codes compared) using a Bonferroni correction. Statistical analysis was performed using SAS version 9.2 (SAS, Cary, NC) and Prism ver. 5.0 (Graphpad Software).

## Results

A total of 30,840 CM hospitalizations were documented over the 13 year period, with a total of 1827 hospitalizations recorded in the most current year (2009). Of those, 24,151 (79.4%) were associated with HIV and 6,689 (21.6%) were not associated with an ICD-9-CM code for HIV/AIDS (042). Based on state population data from the U.S. census in the mid-study year 2003, our analysis included approximately 53.5% of the country’s population during that period. Extrapolating our data to a US population of 307 million in the last study year (2009), we estimated approximately 3,400 hospitalizations associated with CM annually in the US for the most current year for which data was available.

**Figure 1:**
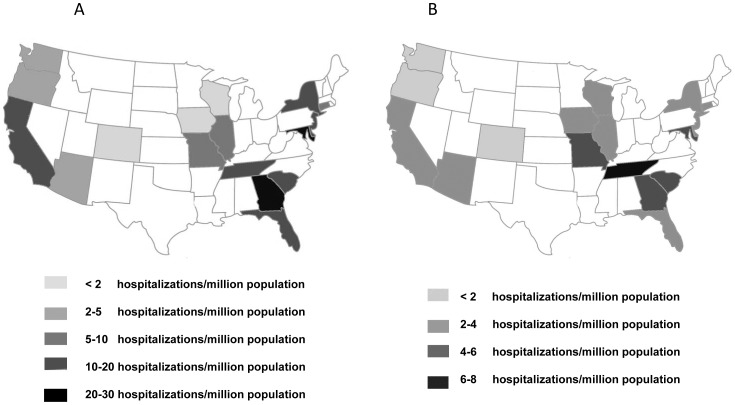
Geographic incidence of CM. A) Map of the US showing, highlighted, states reporting to ARHQ continuously during the study period and their incidence of CM in HIV-infected and B) HIV-uninfected associated hospitalizations per million population.

Rates of HIV-related CM were higher in states with a higher prevalence of HIV infection such as NY, Florida, Maryland and Georgia (Fig. 1A), although high rates of hospitalizations were also evident in the southeastern U.S. (average of 19.4 hospitalizations per million population in TN, GA, SC and FL) with up to 33 hospitalizations per million population in GA. This geographic trend was also evident in non-HIV-related hospitalizations with higher rates in the southeastern states of TN, GA and SC and up to 7.6 hospitalizations per million population in TN (Fig. 1B).

### Trends of Total Hospitalizations

**Figure 2:**
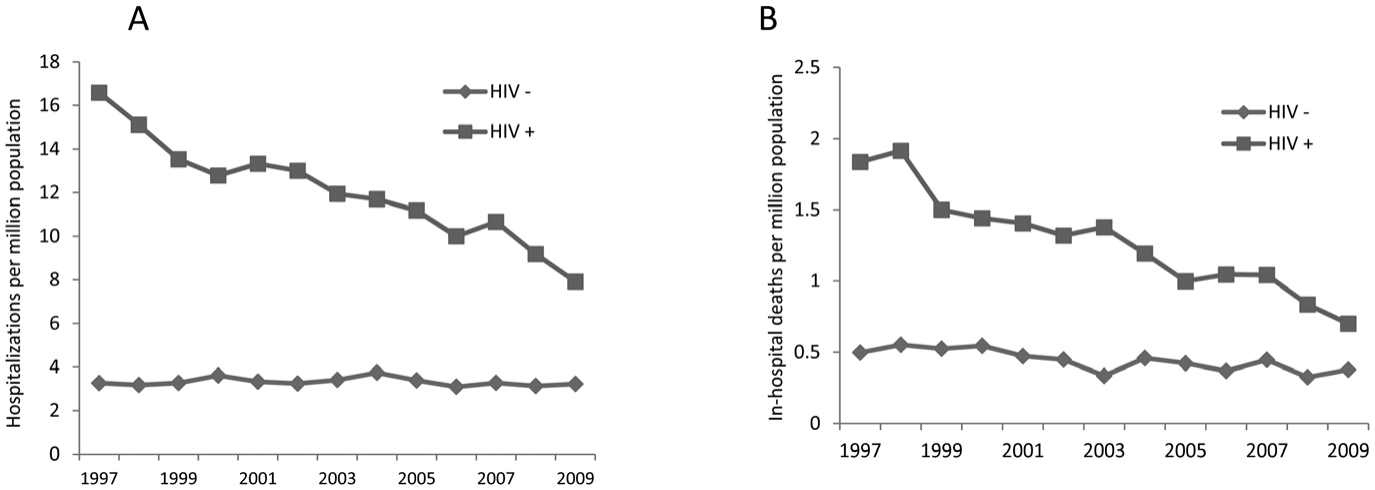
Hospitalizations for CM in the US. A) Hospitalization for CM per million population in HIV-infected and HIV-uninfected patients. B) In hospital mortality per million population in HIV-infected and HIV-uninfected patients.

CM hospitalizations decreased in the HIV-infected patient population during the study period with a high of 16.6 hospitalizations/million total population in 1997 to 7.7 in 2009 (53.6% decline) with the annual percentage change (APC) showing a significant decline of -5.8% (95% CI:-6.5, -5.0) over the time period (Figure 2A). This is comparable to a 53% decline in hospitalization rates due to pneumocystis pneumonia identified from the HCUP database [14] in 11 of the 18 states reporting to this database over the same period (ICD9 code: 136.3). Total hospitalizations among HIV-uninfected individuals also decreased over the study period from 756 hospitalizations per million population in 1997 to 639 hospitalizations/million population in 2009 (APC: -1.7; 95% CI:-2.2,-1.2), which was a slower rate than CM-associated admissions, suggesting that the decrease in CM was not solely due to the overall decrease in HIV hospitalizations. The incidence of non-HIV CM-associated hospitalizations also declined slightly during the study period but at a rate slower than that of HIV-associated CM (Fig. 2A; APC: -1.2; CI-2.0, -0.4). The proportion of non-HIV hospitalizations among all CM hospitalizations thus increased during the study period from 16% in 1997 to approximately 29% during the most recent reporting period. Men predominated among persons hospitalized with CM, in both HIV-infected (78.2%, p<0.0001) as well as HIV-uninfected populations (67.6%, p<0.0001). In the HIV-infected group, rates of hospitalization per million total population peaked in the 31–40 age range, similar to that for the underlying HIV-infected population. In contrast, the highest rates of hospitalizations for CM in the HIV-uninfected group were shifted towards older age groups with a peak seen in the 51–60 age group.

### In-hospital Mortality

We identified a total of 3,440 CM-associated deaths over the 13 year study period with 338 deaths from CM in the first study year, decreasing to 177 deaths in 2009, with an extrapolated total estimate in the US of 330 deaths in 2009. The incidence of in-hospital mortality decreased over the study period in both the HIV-infected (APC:-7.8%;CI:-8.8,-6.7) and HIV-uninfected patients (APC:-4.4;CI:-5.9,-2.8) (Fig. 2B), although a higher proportion of CM hospitalizations ended in death among the HIV-uninfected than among the HIV-infected patients (13.3% vs. 10.5%; p<0.0001).

### Co-morbidities of HIV-Uninfected Patients

Using the SID database, we calculated relative prevalence (RP) of co-morbid conditions, comparing the proportion of hospitalizations with selected comorbid conditions among those with CM to those without. A number of well-known sequelae of CM were identified as significant after adjustment for multivariable comparisons including hydrocephalus (RP: 51) [15] and acute/chronic renal failure (RP: 5.8 and 1.6, respectively), the latter likely due to the use of renal-toxic amphotericin B preparations. Co-morbidities that could represent risk factors for CM included hematopoietic tumors (RP: 9.0), and a variety of conditions associated with immunosuppression including leukopenia (RP: 5.4), sarcoidosis (RP: 9.6), T cell defects (RP: 88), hypogammaglobulinemia (RP: 36), combined B- and T-cell defects (59), and Cushing syndrome (RP: 25). In addition, relationships were also evident with a number of solid organ transplant populations that require immunosuppressant medications, including kidney (RP: 36) and pancreatic (RP: 51). Furthermore, relationships were evident with a variety of rheumatologic diseases including dermatomyositis (RP: 17) and systemic lupus erythematosis (5.4), which could be related to steroid use, known to be a risk factor for CM [16]. Interestingly, a number of codes related to liver disease: liver failure (RP: 6.1), autoimmune hepatitis (RP: 10) and liver cirrhosis (RP: 2.8), were frequent co-morbidities and significantly associated with CM. In addition, co-infections causing viral hepatitis (RP: 2.6) and other fungal infections including oral and invasive candidiasis (RP: 4.9 and 7.3, respectively) as well as diseases caused by intracellular pathogens such as mycobacterial diseases (RP: 17) were also more common among patients hospitalized with CM compared with all patients.

### Financial Burden of CM

The median cost of CM for HIV-uninfected patients was estimated to be $16,803 and $15,708 for HIV-infected patients. This includes only hospitalization costs and does not include outpatient, rehabilitation or lost income estimates. In addition, highest hospitalization costs were found in NY, CT, NJ and MD with an average cost of $17,200 to a low of $12,100 per hospitalization in WA and OR. Hospitalizations were also higher in patients who did not survive, vs. those who did survive ($21,000 vs. $14,400; p<0.001). Based on an estimated number of hospitalizations of 3400 for CM, approximately USD $54 million were spent per year on these direct hospitalization-associated costs associated with CM in the U.S. in 2009.

## Discussion

Here we present nationally-representative population-based estimates for incidence of CM hospitalizations over a 13-year period. These findings from the SID represent the largest epidemiological study of CM in the U.S to include both HIV-infected and uninfected patients representing data from approximately 200 million hospital admissions from both academic and community hospitals. We found a declining incidence of CM hospitalizations associated with HIV as reported earlier [17], and in a more recent single center study at Duke University [9], with a stable incidence of CM among non-HIV associated CM hospitalizations, leading to a relative increase in the proportion of non-HIV CM. This decline was similar to the predominately HIV-related opportunistic infection, pneumocystis pneumonia, using a similar HCUP database [14]. Widespread availability of ART beginning in 1997 likely contributed to the decrease in hospitalizations of these opportunistic infections in HIV-infected patients [2]. More prevalent use of antifungal prophylaxis may also have had an impact [18]. In contrast, hospitalizations in HIV-uninfected patients have been more persistent, increasing the representation of HIV- uninfected patients among CM hospitalizations over time. The rates of hospitalizations were particularly high in the S.E. U.S. with a burden of disease that was similar to that of endemic fungi in this region, such as histoplasmosis (16 hospitalizations per 1,000,000 US population), determined earlier using a similar AHRQ-derived database [19]. The significantly higher proportion of hospitalizations among men in HIV-infected patient populations was not surprising because of higher rates of HIV in males. However, the proportion of males was also higher in HIV-uninfected populations, consistent with smaller studies where male gender was a risk factor for cryptococcal disease among immunocompetent individuals [20], [21]. This male predominance of infection parallels other fungal diseases such as pulmonary blastomycosis [22] and could suggest an environmental influence on exposure, especially since *Cryptococcus* is present in bird droppings, decaying wood and soil [23] and likely represents an increased risk of environmental exposure.

In-hospital mortality decreased over the study period for both HIV-infected and HIV-uninfected patients. This improvement is likely multifactorial and may include advancements in supportive care of critically ill hospitalized patients, as well as the development of less toxic formulations of Amphotericin B, allowing more patients to complete therapy [24]. Another possibility is the impact of IDSA treatment guidelines in the US, including aggressive CSF pressure management, which has helped to standardize treatment and promote evidence based practices [25]. However, in-hospital mortality was found to be higher in HIV-uninfected individuals, which confirms a national trend previously reported in more focused studies [16], [26]. While this difference could be due to increased admissions of HIV patients relative to non-HIV infected patients, HIV- uninfected patients with CM may represent a cohort of patients with more comorbidities such as malignancies and organ transplants that may reduce success.

Our study estimated the frequency of co-morbidities for CM-associated hospitalizations of HIV-uninfected patients in the most recent two years that data was available (2008–2009). Co-morbidities disproportionately represented among HIV-uninfected CM patients (Table 1) included conditions which are known to be associated with cryptococcosis such as hematologic malignancies, rheumatologic diseases, renal failure, solid organ transplants, sarcoidosis and other immune deficiencies, particularly those associated with cellular immune defects [27], lending support for our methodology. While many of these co-morbidities may have predisposed patients to cryptococcosis, others, such as renal failure, may be consequences of anti-cryptococcal treatments requiring renal toxic drugs such as Amphotericin B or the result of the disease itself, such as hydrocephalus [28]. Interestingly, we found an association with multiple codes indicative of liver disease including viral hepatitis, liver failure and cirrhosis. Recently, liver disease has been associated with CM in a retrospective study [29] as well as liver insufficiency [9] and has been associated with poor outcome in *C. gattii* disease [30]. This association could be due to the use of concomitant hepatotoxic drugs during hospitalization although reductions in serum complement often observed in liver disease [31] are important in resistance to CM in animal models [32], thus suggesting avenues for future study. In addition, while a number of T cell defects (T cell defect, leukopenia) was strongly associated with CM, which has been correlated with susceptibility to CM [33], other defects such as hypogammaglobulinemia were also highly associated. While this association could represent coding ambiguities or accompanying defects in cellular immunity, previous studies in humans and animals have shown associations of antibody levels and susceptibility to cryptococcosis [34]–[35], thus again, suggesting possible future studies focusing on this question.

The present studies also found that CM is associated with a significant financial burden with HIV-uninfected patients having a greater cost than HIV-infected patients. This relationship was found previously in a study using the Maryland Hospitalization Discharge Data Set (MDHDDS) conducted in 1997 [36]. In addition, hospitalization costs were found to vary in different regions of the country in accordance with regional differences in Medicare reimbursements as recorded by the center for medical services and reported on the Dartmouth Atlas (www.dartmouthatlas.org) which could be due to regional differences in referral patterns or rates of intensive care utilization [37]. Higher costs in the HIV-uninfected patients could also be due to co-morbidities requiring chemotherapy or transplant conditioning. Another possibility may be the longer four to six week duration of intravenous amphotericin B therapy required in this population, which could lead to longer hospitalizations and higher costs related to amphotericin B infusion toxicity [25].

Large administrative databases such as the SID offer unique opportunities to develop nationally representative estimates of infections among hospitalized patients that can complement more detailed single center studies [9]. This approach is particularly important for the study of rare diseases, for which other approaches are not feasible or cost effective. Nonetheless, these approaches do have some limitations. Diagnoses derived from ICD9 codes could be misclassified as they are often abstracted from charts and may be biased by billing concerns. Misclassification using codes is likely to be greatest for diseases that are challenging to diagnose or require empiric therapy often without microbiological diagnoses [38], [39]. However, a diagnosis of CM is typically made from a sterile cerebrospinal sample with the aid of a highly specific and sensitive serum or CSF antigen test [40]. Interestingly, in a recent validation study of ICD-9 codes, while CM was not specifically tested, other meningitidies with a similar microbiological diagnostic algorithm such as meningococcal and pneumoncoccal meningitis were found to have positive predictive values of in excess of 98% [13]. Thus, coding for CM is likely to be highly specific but with low sensitivity, such that the trends and associations observed are likely to be real, although the absolute disease burden is likely underestimated. Undercoding may also be due to clinical diagnostic delays which are especially common in the immunecompetent CM patients [41]. A second limitation was that our study focused on meningitis caused by *Cryptococcus*, such that we have not included in our incidence estimates of other manifestations of cryptococcosis such as lung, bone or localized skin disease which may be particularly true of infections due to *C. gattii* which have high rates of lung disease without meningitis (29). Restrictions in the study to 18 states due to lack of continuous reporting in the remainder may have influenced overall estimated prevalence estimates if there were unrecognized large deviations in prevalence throughout the U.S. Finally, because such data represent hospital discharges rather than individually-identified patients [14], we are unable to identify how many unique hospitalizations may be from the same individual.

In summary, these studies show the persistence of cryptococcosis in the post-ART era, with an increasing burden of the disease in HIV-uninfected individuals. While the overall incidence of cryptococcosis is decreasing, the epidemiology is complex and changing and it remains an important threat, especially in the HIV-uninfected, emphasizing a continued need for more effective control and treatment strategies in the U.S.
